# Development and validation of the Myasthenia Gravis TeleScore (MGTS)

**DOI:** 10.1007/s10072-022-05918-y

**Published:** 2022-02-28

**Authors:** F. Pasqualin, S. V. Guidoni, E. Albertini, M. Ermani, R. Frangiamore, F. Vanoli, C. Antozzi, R. Mantegazza, D. M. Bonifati

**Affiliations:** 1 Unit of Neurology, Ospedale Regionale Ca’ Foncello, 31100 Treviso, Italy; 2 Department of Neuroscience, University of Padova, 35128 Padova, Italy; 3 Istituto Neurologico Carlo Besta, Milan, Italy

**Keywords:** Telemedicine, Teleneurology, MG, Score, COVID-19 era

## Abstract

**Objective:**

The aim of our study was to validate the Myasthenia Gravis TeleScore (MGTS), a scale for the evaluation of MG patients in telemedicine.

**Introduction:**

COVID-19 pandemic has boosted telemedicine in clinical practice. It could be crucial in the care of neurological patients with chronic disease. However, there is a lack of validated disease-specific tools to evaluate MG patients in telemedicine.

**Methods:**

The MGTS included ten items divided in four districts: ocular, generalized muscular strength, bulbar, and respiratory. Patients were assessed with two different scales: the MGTS and the INCB-MG chosen as a reference from which MGTS was partially derived. Visit in presence with INCB-MG and televisit with MGTS were performed consecutively. Televisit was conducted by another neurologist between two rooms. A blind method was adopted. The strength of correlation was determined by the correlation coefficient (*r*); analysis of covariance (ANOVA—Kruskal–Wallis test) was used to compare subgroups. Significance was set to *p* < 0.05.

**Results:**

One hundred thirty-one patients were included in the study, 71 females and 60 males. The Spearman correlation coefficient between the INCB-MG scale and the MGTS was 0.825 (*p* < 0.001), indicating a very strong correlation between them. Different items showed different correlations from low to high (0.32 to 0.80). As expected, correlation was lower between items with different evaluation modality (anamnestic vs clinical).

**Discussion:**

The MGTS demonstrated a good correlation with INCB-MG, reliability and construct validity.

**Supplementary Information:**

The online version contains supplementary material available at 10.1007/s10072-022-05918-y.

## Introduction

COVID-19 pandemic and restriction to people mobility have boosted telemedicine in medical clinical practice. Until now telemedicine use in neurology was mainly limited to telestroke, in order to bring thrombolysis to rural and unserved areas [1]. However, its use in chronic neurological diseases was more scattered.

The need for “social distancing” during the COVID-19 pandemic boosted the number of teleneurology visits, highlighting the potentiality of this instrument when dealing with fragile patients with chronic diseases.

Roy et al. considered how academic institutions have responded to the present need [2].

Some works have explored the possibility to adapt face to face visit to telemedicine. Grossmann et al. formulated a teleneurology version of neurological evaluation [3].

Hatcher-Martin and his group review evidence-based data on the ability of telemedicine in different neurological subspecialties [4]. Multiple studies have demonstrated favorable results in terms of non-inferiority and patients’ satisfaction. This is easier when the visit is mainly conducted with an interview (Mini Mental and RUDAS for dementia, epilepsia follow-up, drug adjustment and revisions) but it is also possible using standard specific score in a teleneurology modality (i.e., Unified Parkinson’s Disease Rating Scale, Unified Huntington, and the Abnormal Movements Rating Scales) [5, 6].

Several experiences have been reported since COVID-19 started but with non uniform modalities or on single disorder.

Treviso Ca Foncello Hospital group demonstrated feasibility and effectiveness of virtual visits in a large group of patients with different common chronic neurological disorders even in older age [7, 8].

In Malaga analogously telemedicine use was reported in neuromuscular disease with a high rate of success allowing continuative care to fragile patients [9].

Ricciardi et al. made some suggestions for MG patients’ evaluation [10] while Menon et al. evaluate a virtual MG Impairment Index through telephone consultation [11]; however, there is a lack of specific disease tools to evaluate clinically patients in an homogenous and objective way, especially for neuromuscular diseases. Some suggestions on how to evaluate MG patients in telemedicine have been given and a score proposed [12].

The aim of this study was to validate the MGTS score, a telemedicine score that was developed in the first phase of COVID-19 pandemic in order to follow up myasthenia gravis patients despite mobility restrictions.

## Methods

### Item selection and telescore construction

The study included a preliminary phase of item ideation. We reviewed the literature to incorporate items from available measures. Several existing and validated scores were taken into account (ADL, MGC, INCB-MG, MGII, MGDIS, QOL15, QMG). Items from measures identified in the search were reviewed for content and validity, reliability, and responsiveness and those that could be replicated in a telemedicine visit were selected.

Scores that evaluate disability or quality of life were not the aim of our work and were then excluded. After discussion between Treviso and Besta MG groups (all authors), INCB-MG was taken as reference due to its clinical approach, easy to translate in telemedicine, and common use in the two centers.

Like the INCB-MG, the MGTS includes ten items divided in four districts: ocular, generalized muscular strength, bulbar, and respiratory (Table 1). Each item of the scale is graded differently, and a different weight has been arbitrarily chosen for the 4 different areas of clinical involvement (e.g., bulbar and respiratory areas are given greater weight than generalized and ocular ones).

**Table 1:** Comparison INCB and MGTS

	INCB	MGTS	
Ocular level			
0	Normal	0	Normal
1	Diplopia in 1 or 2 cardinal directions, unilateral ptosis	1	Diplopia in 1 or 2 cardinal directions, unilateral ptosis
2	Diplopia in primary position or diplopia in bilateral direction	2	Diplopia in primary position or diplopia in bilateral direction
3	Ophthalmoplegia	3	Ophthalmoplegia
Generalized level			
1) Facial muscles			
0	Normal	0	Normal
10	Orbicularis oculi and/or oris weak but can overcome outside resistance and/or snarl smile	2	Does not perform Souques sign and/or protrudes, and/or whistles
20	Orbicularis oculi and/or oris weak and cannot overcome outside resistance	4	Closes the eye rim but does not tighten, show sign of a smile
30	Lagophthalmos and/or orbicularis oculi/oris plegia	6	Plegia
2) Anterior head/neck flexor muscles			
0	Normal	0	Normal: strongly push with the neck against his hand
10	Weak against resistance	2	Weak against resistance
20	Weak without resistance	4	Weak without resistance (engages accessory muscles and does not push properly)
30	Unable to lift the head	6	Weak without resistance/drop head
3) Abdominal muscles			–
0	Trunk flexion with hands clasped behind the head		–
10	Trunk flexion with forearms extended forward Inability to curl trunk		–
20	Raises shoulder with limbs outstretched		–
30	Inability to curl trunk		–
4) Deltoid muscles			Impairment of ability to brush teeth or comb hair
0	Normal	0	None
10	Weak against resistance	2	Extra effort but no rest period needed
20	Weak without resistance	4	Rest periods needed
30	Unable to abduct upper limbs	6	Cannot do one of these functions
5) Lower extremity muscles			
0	≥ 15 squats	0	≥ 15 squats
10	< 15 squats	2	< 15 squats
20	Able to rise from a normal chair	4	Able to rise from a normal chair
30	Unable to rise from a normal chair	6	Unable to rise from a normal chair
Bulbar level			
1) Chewing			
0	Normal strength of masseter muscle	0	Normal
1000	Weakness of masseters against resistance	8	Fatigue with food
2000	Jaw drop	16	Tube feeding
2) Tongue			
0	Normal	0	Normal
1000	Inability to press the tip against the cheek and/or inability to curl the tongue and reach the upper lip frenulum	8	Inability to press the tip against the cheek and/or inability to curl the tongue and reach the upper lip frenulum
2000	Inability to protrude the tongue	16	Inability to protrude the tongue
3) Phonation			Ask the patient to count till 50
0	Normal	0	Normal
1000	Slight nasal voice	8	Slight nasal voice (30–49)
2000	Severe nasal voice, speech still intelligible	16	Severe nasal voice, speech still intelligible (10–29)
3000	Speech difficult to understand	24	Speech difficult to understand
4) Swallowing			
0	Normal	0	Normal
1000	Dysphagia and/or necessity for soft foods	12	Dysphagia and/or necessity for soft foods
2000	Impossible, tube feeding	24	Impossible, tube feeding
Respiratory level			
0	Normal	0	Normal
200,000	Shortness of breath on exertion	12	Shortness of breath on exertion
300,000	Shortness of breath at rest	24	Shortness of breath at rest
400,000	Mechanical ventilation	36	Mechanical ventilation
Total INCB MG score			
Fatigability			
Upper limbs (seconds) [max 120]			
Lower limbs (seconds) [max 60]			–
Total fatigability			

When possible a clinical approach was preferred. Otherwise the item was adapted to an anamnestic inquiry to the patient. Indeed for the items about chewing and deltoid, clinical exploration was not possible. Thus, these items were adapted from the MGC and ADL, respectively, that investigate them in an anamnestic way.

We expected a lower correlation between MGTS and INCB in the items with different approaches (anamnestic vs clinical) and higher between those items with similar one.

We expected a low correlation in fatigability due to a different posture in the evaluation: in the INCB the patient is supine with 45° between arms and body, while in the MGTS the patients sit in front of the monitor with arms parallel to the ground.

Patients were assessed with two different scales: the MGTS and the INCB-MG chosen as reference. The abdominal muscle item was not included because of its low correlation with MG status and for the difficulty to explore it in a common telemedicine visit. Indeed, from our previous experience [7] the patient during a televisit is sitting in front of the webcam and it is not easy to ask him to lie down.

A blind method was adopted: the first neurologist conducted a follow-up visit in presence and collected the INCB-MG evaluation. Soon after another physician conducted the second evaluation assessing the MGTS score in a televisit modality between two rooms in the clinic. Google Meet platform was used for the teleneurology visit.

Patients were asked to not take their anticholinesterase medication before the visit.

### Sample size

To calculate the sample size, we used the minimal correlation expected in the construct validity studies. For a minimal correlation of *r* = 0.4, with alpha = 0.05 and 90% power a minimum of 62 patients are needed. COSMIN recommends a minimum of 100 patients. We recruited more (131) to get better understanding of the performance across the disease spectrum.

### Population

One hundred thirty-one patients were included in the study. We classified them in the following subgroups: ocular (symptoms strictly ocular for at least 2 years from onset), early onset MG (generalized anti-acetylcholine positive with age at onset < 50 years), late onset MG (generalized anti-acetylcholine positive with age at onset ≥ 50 years), anti-MuSK MG, double seronegative, and thymoma-associated MG. Demographic and clinical characteristics of each patient were recorded. Outcome to treatment was registered with the MGFA-PIS and with the MGSTI.

The study was conducted in accordance with the ethical standards of the institutional and national research committee and with the 1964 Helsinki Declaration and its later amendments or comparable ethical standards. The local ethical committee of ULSS2—Ca’ Foncello Hospital approved this study and written informed consent was obtained.

### Statistical analysis

For statistical comparison of the 2 MG scales, we recorded the INCB-MG and the MGTS scale in units.

Variables, if possible, were expressed as dichotomous variables. The strength of correlation was determined based on the correlation coefficient (*r*): very high (0.9 to 1.0); high (0.7 to 0.9); moderate (0.5–0.7); low (0.3 to 0.5); and negligible (0.3 to 0.0). These correlations were plotted, and analysis of covariance (ANOVA—Kruskal–Wallis test) was used to compare subgroups. Significance was set to *p* < 0.05.

### Interrater and test–retest reliability

We tested interrater reliability (IRR): two neurologist independently evaluated twenty-six patients in the same televisit between two rooms, blinded to each other’s scores. IRR was tested with the weighted kappas for each item, district and the global scores. There is no universal consensus on the interpretation of kappa, but usually values between 0.6 and 0.8 are considered substantial and 0.8 excellent agreement [13]. Finally, we calculated the standard error of measurement.

## Results

### Characteristics of the sample

One hundred thirty-one patients were included in the study, 71 females and 60 males. The average age was 60.0 ± 14.9 which was higher in males than in females: 67.7 ± 11.9 vs 53.7 ± 14.3, respectively (*p* < 0.0001).

The sample included patients with the following subtypes of MG: 20 with ocular MG (3 females, 17 males), 30 EOMG (25 females, 5 males), 37 LOMG (14 females, 23 males), 15 thymoma-associated MG (9 females, 6 males), 12 anti-MuSK positive (10 females, 2 males), and 17 double seronegative (10 females, 7 males).

The mean age at the onset was 50.2 ± 19.0 and it was higher in males than in females (49.7 ± 16.5 vs 42.0 ± 17.3, *p* < 0.00001). The disease duration was on average 10.2 ± 11.2. It was higher in females than in males (12.2 ± 12.4 vs 8.0 ± 10.0, *p* = 0.012) (Table 2).

**Table 2:** Clinical characteristics of the sample

*N* of patients		Total (131)
Sex female/male		71/60
Average age		60.0 ± 14.9
Average age at onset		50.2 ± 19.0
Disease duration		10.3 ± 11.6
MGFA-PIS at last follow-up	CSR	5 (3.8%)
	PR	21 (16.0%)
	MM-0	6 (4.6%)
	MM-1	16 (12.2%)
	MM-2	7 (5.3%)
	MM-3	25 (19.1%)
	Symptomatic	51 (38.9%)
MGSTI at last follow-up visit	0	18 (13.7%)
	1	26 (19.8%)
	2	32 (24.4%)
	3	4 (3.1%)
	4	37 (28.3%)
	5	14 (10.7%)
	Total	131

The clinical status and outcome at follow-up visit were collected with two scales: MGFA-PIS and MGSTI (Table 2).

**Fig. 1:**
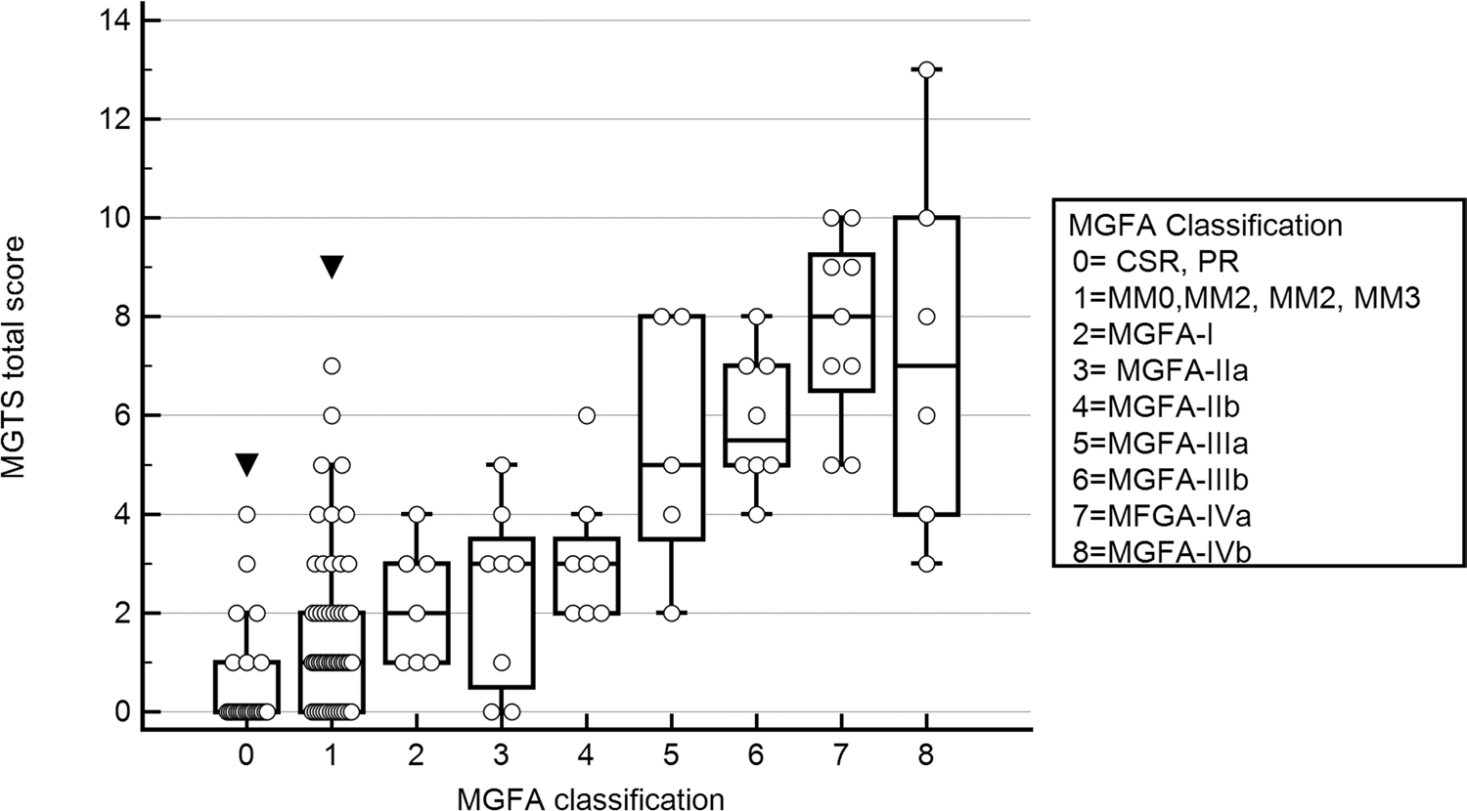
Total scores according to different MGFA classes. Patients in remission had very low total scores and scores increased progressively with higher MGFA class (*p* < 0.000001)

Patients in remission had very low total scores and scores increased progressively with higher MGFA class reflecting clinical status and predominant bulbar or limb weakness (Fig. 1).

### Validity

**Fig. 2:**
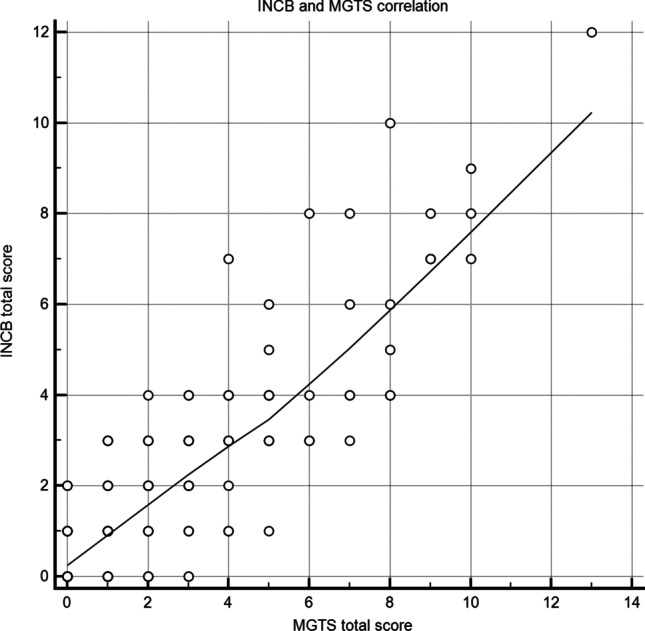
INCB and MGTS correlation. The Spearman correlation coefficient between the INCB-MG scale and the MGTS was 0.825 (*p* < 0.001), indicating a very strong correlation between them

The significance of correlation between MGTS and the reference scale is illustrated in Table 3. The Spearman correlation coefficient between the INCB-MG scale and the MGTS was 0.825 (*p* < 0.001), indicating a very strong correlation between them (Fig. 2).

**Table 3:** Validity

	Pair of variables	Sample size	*R *Spearman MGTS and INCB (*p*)	*p*	95% confidence interval for rho
MGTS TOTAL & INCB TOTAL		131	**0.825**	*p* < 0.0001	0.762 to 0.873
OCULAR	**1 MGTS & INCB-MG**	131	0.645	*p* < 0.0001	0.532 to 0.735
MIMIC	**2.1 MGTS & INCB-MG**	131	0.503	*p* < 0.0001	0.363 to 0.621
NECK	**2.2 MGTS & INCB-MG**	131	0.513	*p* < 0.0001	0.375 to 0.629
DELTOID	**2.3 MGTS-2.4 INCB-MG**	131	0.344*	*p* < 0.0001	0.170 to 0.476
LOWER LIMB	**2.4 MGTS-2.5 INCB-MG**	127	**0.803**	*p* < 0.0001	0.731 to 0.853
CHEWING	**3.1 MGTS-3.1 INCB-MG**	131	*0.329§*	*p* < 0.0001	0.167 to 0.474
TONGUE	**3.2 MGTS-3.2 INCB-MG**	131	0.677	*p* < 0.0001	0.572 to 0.760
PHONATION	**3.3 MGTS-3.3 INCB-MG**	131	0.653	*p* < 0.0001	0.542 to 0.742
SWALLOWING	**3.4 MGTS-3.3 INCB-MG**	131	**0.738**	*p* < 0.0001	0.648 to 0.807
RESPIRATORY	**4 MGTS-4 INCB**	131	**0.749**	*p* < 0.0001	0.662 to 0.816

The strength of correlation between INCB and MGTS was determined based on the correlation coefficient (r): very high (0.9 to 1.0); **high (0.7 to 0.9)**; moderate (0.5–0.7); *low (0.3 to 0.5)*; and negligible (0.3 to 0.0)

^*^Testing the correlation about the DELTOID item between MGTS and ADL (both anamnestic), the *R* Spearman was 0.868 (*p* < 0.0001)

^§^Testing the correlation about the CHEWING item between MGTS and MGC (both anamnestic), the *R* Spearman was 0.775 (*p* < 0.0001)

Deltoid and chewing items showed a lower correlation, even though this was expected due to different methods in the examination. Indeed testing the correlation about the deltoid and the chewing items between MGTS and ADL (both anamnestic), the *R* Spearman was 0.868 (*p* < 0.0001) and 0.775 (*p* < 0.0001), respectively.

Fatigability showed a significant difference between INCB and MGTS (*p* < 0.05).

ICC for the total score was 0.84 (95% CI 0.78 to 0.88).

### Reliability

Twenty-six patients were assessed for interrater reliability for all items and for the global scale and districts. Interobserver reliability for the MGTS scale was *K* 0.894 (95% CI 0.83 to 0.95).

All items had weighted kappa values between 0.61 and 1.00.

These results indicate a very high degree of concordance between the 2 observers.

## Discussion

Our previous experiences showed that telemedicine is a useful instrument in the follow-up of chronic neurological disease. Rosellini et al. showed feasibility and effectiveness of virtual visit in the management of a large group of patients with different common chronic neurological disorders [7].

Up to now few neurological disease-specific scores, for Parkinson, Huntington, and stroke, have been tested in a teleneurology modality.

Recently in MG, Menon et al. evaluate a virtual MG Impairment Index through telephone consultation and compare it with other patients reported outcomes. They showed that virtual MG Impairment Index was an effective measure of disease status in telephone consultations [11]. However, the study was not addressed to evaluate clinically MG patients in an out-patient setting. Some suggestions on how to evaluate MG patients in telemedicine have been given and a score proposed after discussion and unblinded voting at seven MG centers in the USA [12].

The paper reviewed the applicability in telemedicine of some QoL scores and gives some general instruction about how to perform telemedicine consultations in MG and suggests a possible score (MG Core exam scoring) derived from QMG but the score has been not yet validated in a large cohort of MG patients.

The MGTS adds a new instrument to the existing tools for specific neurological disease to use in telemedicine.

A strong correlation between MGTS and INCB-MG scale has been demonstrated and this supports the use of this tool in the clinical evaluation of MG patients.

All the evaluated domains for muscle strength proved to be concordant except muscle fatigability; indeed, the difference can be attributed to a different posture and physical effort as the evaluator is not in presence. A relevant advantage to use MGTS is that, by using it, one can have a comprehensive vision of patient status.

Like in the INCB-MG, the arm endurance test does not go into the total score but in the practice it is useful to evaluate arm fatigability and changes in follow-up visit or televisit.

Overall, the MGTS represents the teleneurology version of the INCB-MG scale, though some items have been adapted from other scores, e.g., MG-ADL, to optimize the examination in telemedicine modality. Indeed some districts and items are better explored with physical examination, while others need anamnestic question. To evaluate chewing for example, history is often superior (less likely to have floor effects) than examining the masseter. This suggests the usefulness of including patients’ reported items to medical exploration, giving a global description of the status of the patient.

From our previous experience [7], the patient during a televisit is sitting in front of the webcam and it is not easy to ask him to lie down so some items proposed by the MG Core exam could not be so easily performed by the patients.

Teleneurology during COVID-19 pandemic has been critically important to follow up chronic patients such as myasthenics and has compensated face-to-face visits cannot be totally replaced by a virtual one. However, chronic disease patients need regular follow-up in order to review examinations or therapeutic adjustment. It is possible that here is where telemedicine will find its role in the common management of chronic neurological patients.

A limit of the study is the sample size that has been reduced for the outbreak of the second wave of the pandemic. Studies assessing the responsiveness and minimal important difference of the MGTS in detecting changes in disease severity in the single patient during follow-up are underway.

Because the score is designed to an out-patient population and it is unlikely to be used in ICU, the last item of the respiratory item (mechanical ventilation) could be substituted by BiPaP use.

In conclusion, we think that the MGTS is a useful instrument in teleneurology, and its future use in everyday management of myasthenic patients should be considered.

## Supplementary Information

Below is the link to the electronic supplementary material.

Supplementary file1 (DOCX 8 KB)

## Data Availability

The data that support the findings of this study are available from the corresponding author upon reasonable request.
