# Supplementary material for: Development and validation of the Myasthenia Gravis TeleScore (MGTS)
Source: Neurol Sci. 2022 Feb 28;43(7):4503–9. doi: 10.1007/s10072-022-05918-y (PMC8883006; doi:10.1007/s10072-022-05918-y)
Supplement: Supplementary file 1 — Supplementary file1 (DOCX 8 KB) [file 10072_2022_5918_MOESM1_ESM.docx]

Supplemental materials

| ITEM | SAMPLE SIZE | WEIGHTED KAPPA | 95% CI |
| --- | --- | --- | --- |
| OCULAR | 26 | 0.89 | 0.74 to 1 |
| MIMIC | 26 | 0.61 | 0.44 to 0.79 |
| NECK | 26 | 1 | 1 to 1 |
| DELTOID | 26 | 0.95 | 0.86 to 1 |
| LOWER LIMB | 26 | 1 | 1 to 1 |
| CHEWING | 26 | 1 | 1 to 1 |
| TONGUE | 26 | 1 | 1 to 1 |
| PHONATION | 26 | 0.83 | 0.62 to 1 |
| SWALLOWING | 26 | 1 | 1 to 1 |
| RESPIRATORY | 26 | 1 | 1 to 1 |
| tot ocular items | 26 | 0.88 | 0.72 to 1 |
| tot general strenght item | 26 | 0.88 | 0.75 to 0.93 |
| tot bulbar item | 26 | 0.94 | 0.89 to 1 |
| tot respiratory | 26 | 1 | 1 to 1 |
| total MGTS score | 26 | 0.89 | 0.83 to 0.95 |
